# Role of Receptor for Advanced Glycation End-Products in Endometrial Cancer: A Review

**DOI:** 10.3390/cancers16183192

**Published:** 2024-09-19

**Authors:** Kamila Zglejc-Waszak, Marcin Jozwik, Michael Thoene, Joanna Wojtkiewicz

**Affiliations:** 1 Department of Anatomy, Faculty of Medicine, Collegium Medicum, University of Warmia and Mazury in Olsztyn, 10-082 Olsztyn, Poland; 2 Department of Gynecology and Obstetrics, Collegium Medicum, University of Warmia and Mazury in Olsztyn, 10-045 Olsztyn, Poland; 3 Department of Medical Biology, Faculty of Health Sciences, University of Warmia and Mazury in Olsztyn, Żołnierska 14C Str., 10-561 Olsztyn, Poland; michael.thoene@uwm.edu.pl; 4 Department of Human Physiology and Pathophysiology, Faculty of Medicine, Collegium Medicum, University of Warmia and Mazury in Olsztyn, 10-082 Olsztyn, Poland

**Keywords:** endometrial cancer, glycation, RAGE, Diaph1, DNA methylation

## Abstract

**Simple Summary:**

The RAGE signaling pathway is associated with many chronic disorders. One of them may be endometrial cancer (EC). EC is one of the most common gynecologic cancers among women. The aim of our considerations was to decipher the role of RAGE and its ligands in the progression of EC. We confirmed that disruptions in the RAGE signaling pathways may be a reason for EC progression. The cross-talk between RAGE and Diaph1 plays a crucial role in downstream signaling pathways during EC progression. However, we assume that DNA methylation may regulate the activity of the RAGE signaling pathways in the endometrium. Thus, DNA methylation may create an epigenetic memory that may trigger perturbations in the endometrium.

**Abstract:**

Endometrial cancer (EC) is the most common gynecological malignancy. EC is associated with metabolic disorders that may promote non-enzymatic glycation and activate the receptor for advanced glycation end-products (RAGE) signaling pathways. Thus, we assumed that RAGE and its ligands may contribute to EC. Of particular interest is the interaction between diaphanous-related formin 1 (Diaph1) and RAGE during the progression of human cancers. Diaph1 is engaged in the proper organization of actin cytoskeletal dynamics, which is crucial in cancer invasion, metastasis, angiogenesis, and axonogenesis. However, the detailed molecular role of RAGE in EC remains uncertain. In this review, we discuss epigenetic factors that may play a key role in the RAGE-dependent endometrial pathology. We propose that DNA methylation may regulate the activity of the RAGE pathway in the uterus. The accumulation of negative external factors, such as hyperglycemia, inflammation, and oxidative stress, may interfere with the DNA methylation process. Therefore, further research should take into account the role of epigenetic mechanisms in EC progression.

## 1. Introduction

Endometrium is an important tissue in pregnancy development, essential for reproductive success [1,2]. Yet, endometrium is also a site of the most common malignancy of the female reproductive system, endometrial cancer (EC) [3,4,5]. Many novel aspects of the physiology and pathology of the endometrium are currently under study [6,7].

The inspiration to write a review were the results of NGS studies, which revealed the association of metabolic changes with the development of EC in women [4]. In this review, we aimed to summarize the current knowledge on the role of the receptor for advanced glycation end-products (RAGE) in EC. Our previous studies have indicated that RAGE and its ligands are involved in diabetic/metabolic complications, as well as in neurodegenerative diseases [8,9,10,11,12,13,14]. We demonstrated that the RAGE signaling pathway may be activated in uterine tissues during the estrous cycle [6]. Zheng et al. [5] showed a stimulative effect of RAGE on the formation of microvessels in EC. This can be seen as an analogy to diabetic disorders, like retinopathy [15]. Previous studies have indicated that RAGE signaling may be associated with renal cell carcinoma, as well as prostate, lung, breast, and colorectal cancers [5,16,17,18]. Therefore, we analyzed the role of RAGE as a potential prognostic protein of uterine carcinogenesis. We assumed that the RAGE signaling pathway may be involved in EC development.

The insights on the role of RAGE signaling in EC provided in this review have been based on the literature as well as on the results of our previous studies. We describe recent advances and current theories on the etiology of EC, highlighting the function of the RAGE signaling system in endometrial pathology. As EC is a broad topic with new information coming out almost every week, we, therefore, focused our review on aberrant RAGE-mediated signaling in EC to formulate a new hypothesis. We believe that limiting the scope has allowed us to focus on how the interaction between RAGE and its ligand may play a pivotal role in endometrial complications in animal models and human patients and if understanding this pathway can uncover targets for safe and effective therapeutic intervention for EC.

## 2. Molecular Dialogue between the RAGE Signaling Pathway and Endometrial Pathology

RAGE is a multi-ligand receptor widely expressed in good health and in embryogenesis, as well as in health disorders [8,9,10,11,12,13,14,19,20]. The consequential discovery that RAGE was a multi-ligand receptor set the stage for a better understanding of the biology of RAGE in homeostasis and in disease. We observed that the expression of RAGE was elevated during neurodegenerative diseases and in diabetes [8,9,10,11,12,13,14,20,21]. We noted that the RAGE signaling pathway is activated in the uterus during the estrous cycle [6]. It has, therefore, been difficult to separate the physiological role of RAGE from its pathological influence on the development of EC. Nowadays, EC is the most common cancer of female reproductive organs, and new insights into the RAGE signaling pathway may find novel indications for the early detection and treatment of EC.

Approximately 40% of EC cases are associated with acquired metabolic disorders, such as obesity and diabetes [4,22,23]. Nevertheless, Sidorkiewicz and co-workers [4] identified genes associated with metabolic status in EC. Transcriptomic studies showed that *solute carrier family 7 member 11* (*SLC7A11*), *solute carrier family 7 member 5* (*SLC7A5*), *Runt-related transcription factor 1* (*RUNX1*), *laminin subunit alpha 4* (*LAMA4*), *collagen type VI alpha 3 chain* (*COL6A3*), *phosphoinositide-dependent kinase-1* (*PDK1*), *cyclin A1* (*CCNA1*), *enolase 1* (*ENO1*), *pyruvate kinase muscle isozyme* (*PKM*), *nuclear receptor subfamily 2 group F member 1* (*NR2F1*), and *N-acetylated alpha-linked acidic dipeptidase 2* (*NAALAD2*) are directly associated with the KEGG signaling pathway, the central carbon metabolism in cancer (hsa05230) [4].

**Figure 1:**
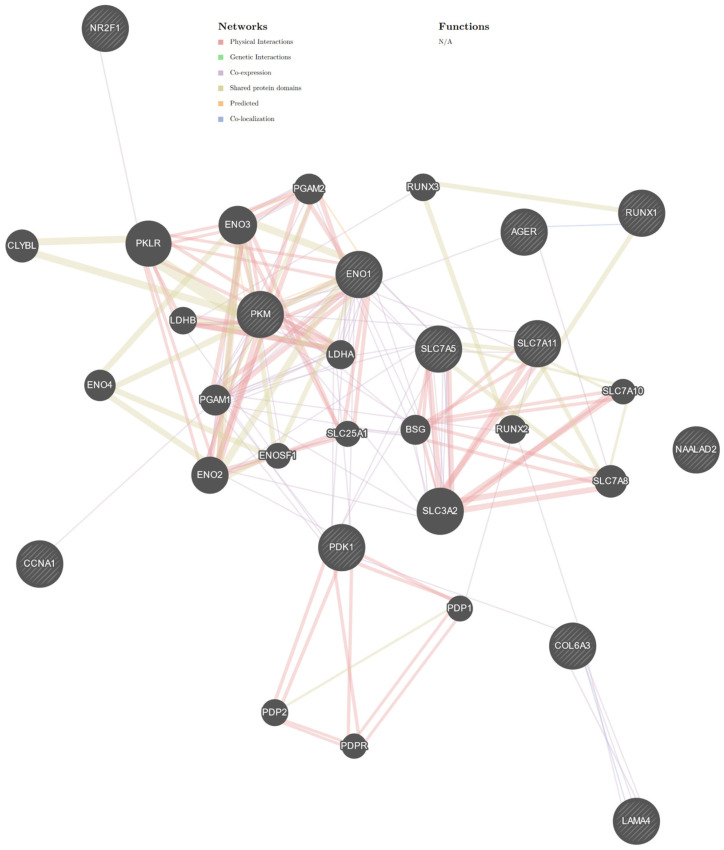
Interaction network constructed for genes involved in central carbon metabolism in cancer (hsa05230) [4] limited to *Homo sapiens* (https://genemania.org/) [24]. The GeneMANIA is a prediction server used for biological network integration for gene prioritization and predicting gene function. The server additionally adds genes based on known interactions. Additional genes are represented by gray, unstriped circles. Selected genes are represented by circles with stripes. The colors of the line suggest the type of interaction, that is, red—physical interactions; purple—co-expression; orange—predicted; blue—co-localization; light blue—pathway; green—genetic interactions; olive—shared protein domains. Interactors are shown with the official gene symbol. *NAALAD2* is the only gene found outside the network. Most studies indicate that *NAALAD2* is involved in prostate cancer. Sidorkiewicz et al. [4] may be the first to have correlated this gene with endometrial cancer (EC). However, the expression of *NAALAD2* was decreased in the endometrium during cancer [4]. Abbreviations: *AGER*—the gene encoding receptor advanced glycation end-products; *ENO2*—enolase 2; *ENO1*—enolase 1; *PKM*—pyruvate kinase isozymes M1/M2; *PGAM2*—phosphoglycerate mutase 2; *ENO3*—enolase 3; *CCNA1*—cyclin A1; *ENO4*—enolase 4; *ENOSF1*—enolase superfamily member 1; *CLYBL*—citramalyl-CoA lyase; *NAALAD2*—N-acetylated alpha-linked acidic dipeptidase 2; *LAMA4*—laminin subunit alpha 4; *COL6A3*—collagen type VI alpha 3 chain; *NR2F1*—nuclear receptor subfamily 2 group F member 1; *PDP2*—pyruvate dehydrogenase phosphatase catalytic subunit 2; *PDPR*—pyruvate dehydrogenase phosphatase regulatory subunit; *PDP1*—pyruvate dehydrogenase phosphatase catalytic subunit 1; *PDK1*—pyruvate dehydrogenase kinase 1; *RUNX1*—RUNX family transcription factor 1; *RUNX2*—RUNX family transcription factor 2; *RUNX3*—RUNX family transcription factor 3; *CCNA1*—cyclin A1; *LDHA*—lactate dehydrogenase A; *LDHB*—lactate dehydrogenase B; BSG—basigin; *PKLR*—pyruvate kinase L/R; *PGAM1*—phosphoglycerate mutase 1; *SLC25A1*—solute carrier family 25 member 1; *SLC7A5*—solute carrier family 7 member 5; *SLC7A8*—solute carrier family 7 member 8; *SLC7A11*—solute carrier family 7 member 11. N/A—not applicable. See File S1.

The GeneMANIA [24] analysis of selected genes, including *SLC7A11*, *SLC7A5*, *RUNX1*, *LAMA4*, *COL6A3*, *PDK1*, *CCNA1*, *ENO1*, *PKM*, *NR2F1*, *NAALAD2* [4], and *AGER* (the gene encoding RAGE), revealed that only *NAALAD2* was not connected to one network (Figure 1). The GeneMANIA network comprises 32 genes (20 related genes) and consists of genes related to multiple biological functions (Table S1). The analysis revealed that the selected genes were mainly involved in metabolic functions (Table 1). Moreover, we observed that *AGER* directly interacts with *PKM* and *SLC7A8* (a co-expression interaction) and *RUNX1* (a co-localization interaction, Figure 1).

According to The Cancer Genome Atlas, 20,000 primary cancers have been identified in this novel look at the etiology of cancer. A new classification of EC by The Cancer Genome Atlas revealed that metabolic reprogramming may be one of the causes of cancer progression in women [4,25]. Studies have indicated malfunctions in cell metabolism driven by oncogenes [25]. Therefore, malfunctions in metabolic pathways may be a characteristic feature of cancer [26,27]. However, so far only the Warburg effect has been focused on as a metabolic cause of EC [28,29,30]. In this review, we would like to demonstrate that RAGE signaling pathways may play a crucial role in the progression of metabolic cancer in the female reproductive tract.

Previous studies have shown that mTOR and PI3K/AKT metabolic signaling pathways are associated with EC progression [4,25,31]. Nevertheless, Ramasubbu and Devi Rajeswari [32] indicated that RAGE may inhibit the mammalian target of rapamycin (mTOR) and phosphatidyl inositol 3-kinase-AKT (PI3K/AKT) signaling pathways during diabetes. Thus, RAGE signaling pathways may play a role in cancer treatments.

Nevertheless, mTOR and PI3K/AKT signaling pathways are involved in cell proliferation and microvessel formation in EC [5]. Moreover, RAGE during metabolic disorders is associated with new microvessel formation [33].

Zheng and co-workers [5] revealed that RAGE may be associated as a potential regulatory factor of microvessel formation in EC. Studies have indicated that a similar phenomenon has been observed in other cancer types [34,35]. Thus, RAGE signaling pathways, as well as mTOR and PI3K/AKT signaling pathways, may be involved in microvessel formation during EC growth [16,34,35]. Studies have indicated that RAGE ligands may also play a key role in the progression of microvessel formation during EC [17,29]. Overall, RAGE and its ligands are involved in metabolic complications as well as microvessel formation during numerous pathological conditions [8,9,12,13,14,15,33,34,35].

The amount of RAGE protein is elevated in the endometrium during type 1 diabetes, as well as with a high-fat diet [6]. Studies have shown that the amount of RAGE is the highest in EC, particularly in less differentiated tumors [5,36]. Consequently, we may observe the elevated level of advanced glycation end-products (AGEs) in EC [5,36]. Thus, RAGE and its ligands may be associated with progression of EC (https://www.proteinatlas.org/ENSG00000204305-AGER/pathology, accessed on 1 August 2024).

AGEs are formed during the Maillard reaction. The Maillard reaction is a non-enzymatic process in which glucose reacts with protein, i.e., glycation. AGEs are accumulated in our body through high glucose levels or ingestion [37]. The main precursor of AGEs is methylglyoxal (MGO). However, under physiological conditions, glyoxalase 1 (GLO1) is responsible for the detoxification of MGO [8,9,12,38]. Studies have indicated that the amount of AGEs is elevated during pathophysiological conditions such as diabetes, neurological diseases, and cancer [8,9,10,11,12,13,14,15,39,40]. Moreover, reports have shown that AGEs trigger disorders by several mechanisms: (1) AGE intracellular accumulation; (2) disturbances in the proper structure and function of proteins binding to proteins; and (3) activation of the RAGE signaling pathway [8,9,12,13,14,15,39]. Previous studies have indicated that the pathological process in the cell is preceded by an increased amount of RAGE-AGEs [41]. Next, the studies revealed the various forms of stress in the cell during disorders [41]. Our studies revealed the pathological process of RAGE-AGEs in the endometrium during the estrous cycle [6]. Therefore, we may speculate that RAGE-AGEs may be involved in the pathophysiological processes of EC [42,43].

## 3. RAGE and Its Ligands in Endometrial Cancer (EC)

**Figure 2:**
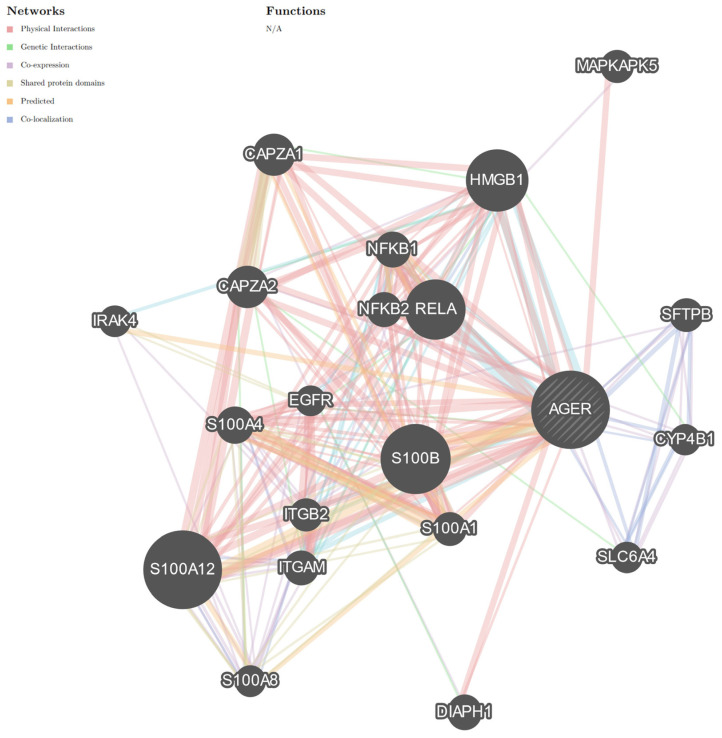
Graph representing the interaction network of *AGER* (the gene encoding RAGE) with genes encoding RAGE ligands. *HMGB1*, *S100B*, and *Diaph1* are the most well-known RAGE ligands. The interaction of *AGER/RAGE* with *HMGB1*, *S100B*, and *Diaph1* may be crucial for EC progression. The interaction network includes the cancer-related factors *NFKB1*, *NFKB2*, and *EGFR*. The colors of the line suggest the type of interaction, that is, red—physical interactions; purple—co-expression; orange—predicted; blue—co-localization; light blue—pathway; green—genetic interactions; olive—shared protein domains. Limited to *Homo sapiens*. Abbreviations: HMGB1—high-mobility group box 1; *S100B*—S100 calcium-binding protein B; *Diaph1*—diaphanous-related formin 1; *MAPKAPK5*—MAPK-activated protein kinase 5; *CAPZA1*—capping actin protein of muscle z-line subunit alpha 1; *CAPZA2*—capping actin protein of muscle z-line subunit alpha 2; *IRAK4*—interleukin 1 receptor-associated kinase 4; *NFKB2*—nuclear factor kappa B subunit 2; *NFKB1*—nuclear factor kappa B subunit 1; *RELA*—RELA proto-oncogene, NFKB subunit; *EGFR*—epidermal growth factor receptor; *S100A4*—S100 calcium-binding protein A4; *ITGB2*—integrin subunit beta 2; *SFTPB*—surfactant protein B; *CYP4B1*—cytochrome P450 family 4 subfamily B member 1; *S100A1*—S100 calcium-binding protein A1; *SLC6A4*—solute carrier family 6 member 4; *ITGAM*—integrin alpha M; *S100A8*—S100 calcium-binding protein A8; *S100A12*—S100 calcium-binding protein A12. See File S2. N/A—not applicable. The interaction network using GeneMANIA was also created in the publication Zglejc-Waszak et al. [8]. These are not the same interaction networks as in the previous publication [8]. The similarity may result from the databases used by the GeneMANIA server.

Studies have indicated that patients with diabetes are at high risk of cancer progression [4,5,36]. Our data revealed that the expression of RAGE and its ligands is elevated in diabetic patients [8,9,12,13,14,15,40,44]. It can therefore be inferred that endometrial carcinogenesis may be related to increased expression of RAGE and its ligands. RAGE binds with a variety of ligands, especially proinflammatory ones, and is engaged with cytoskeletal dynamics [8,9,13,14]. Studies have shown that the amounts of proinflammatory high-mobility group box 1 (HMGB1) and S 100 calcium-binding protein B (S100B) ligands increase in cancer [42,43,45,46]. GeneMANIA analysis indicated that *HMGB1* and *S100B* interact with *AGER* (the gene encoding RAGE) through physical-, predicted-, and co-localized interactions (Figure 2). These analyses revealed another important interaction between *AGER/RAGE* and *diaphanous-related formin 1* (*Diaph1*; Figure 2), but an exact role of these molecules is unclear in EC (https://www.proteinatlas.org/ENSG00000204305-AGER/pathology, accessed on 1 August 2024; https://www.proteinatlas.org/ENSG00000131504-DIAPH1/pathology, accessed on 1 August 2024).

### 3.1. HMGB1

Our studies have indicated that the expression of HMGB1 is elevated in the diabetic sciatic nerve as well as in the spinal cord harvested from SOD1 G93A mice [8,9,10,11,12,13,14,15]. Moreover, further studies revealed that increased expression of HMGB1 is present in Parkinson’s, Alzheimer’s, cardiovascular diseases, stroke, retinopathy, neuropathy, as well as amyotrophic lateral sclerosis [12,47,48,49,50]. Previous studies revealed that the amount of HMGB1 is elevated in the endometrium harvested from type 1 diabetic mice and mice with a high-fat diet [6]. Our studies have shown that HMGB1 is associated with the inflammation process in the uterus [6]. We may suppose that HMGB1 is related to a chronic pathological process that takes place in the endometrium (https://www.proteinatlas.org/ENSG00000189403-HMGB1/pathology, accessed on 1 August 2024). HMGB1 in hormone-related cancer may be a potential therapeutic target [45]. This phenomenon has not been observed in healthy cells [51].

Histologically, EC may be classified into three grades: grade I—well differentiated, with a better prognosis; grade II—moderately differentiated; and grade III—poorly differentiated, with a worse prognosis. Luan et al. [52] revealed that HMGB1 is negatively correlated with the development of endometrial carcinoma and prevents cancer cell invasion and metastasis by inhibiting the process of epithelial-to-mesenchymal transition. Thus, we may suppose that the role of HMGB1 is not significant in EC type II, as type II ECs are poorly differentiated [52]. It should be stressed that HMGB1 is a RAGE ligand, as the RAGE-HMGB1 interaction may have a strong impact on the progression of endometrial carcinoma [42,52]. Studies have indicated that HMGB1 interacts with RAGE in tumor cells [51].

**Figure 3:**
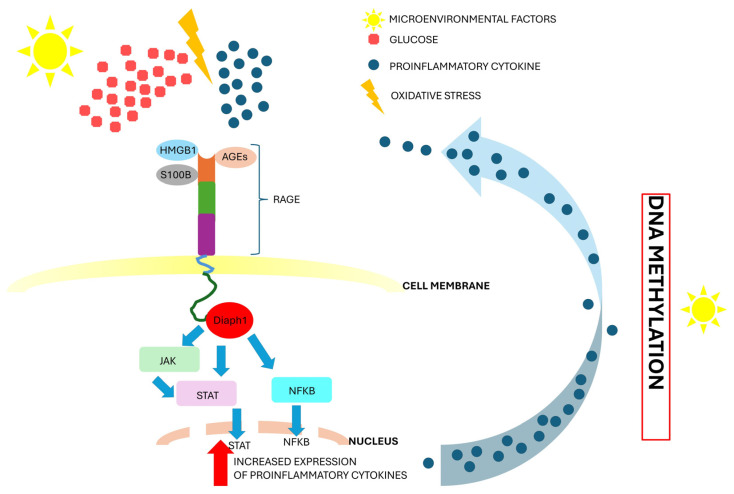
Schematic of the RAGE signaling pathway in EC and potential therapeutic targets. High glucose levels, oxidative stress, and local inflammation activate the RAGE signaling pathway. The V domain (extracellular domain) of RAGE interacts with HMGB1, S100B, and AGEs. HMGB1, S100B, and AGEs are proinflammatory RAGE ligands. Diaph1 interacts with the cytoplasmatic domain. Studies have indicated that Diaph1 may be a crucial player in the downstream RAGE signaling pathway. The signal cascade activates transcription factors (NFKB, STAT) that increase the amount of proinflammatory cytokines. Moreover, inhibiting the RAGE-Diaph1 interaction may contribute to reducing the production of proinflammatory cytokines. However, DNA methylation may have a key impact on RAGE-dependent cell signaling. Local environmental factors, hyperglycemia, oxidative stress, and inflammation may induce malfunctions in DNA methylation and, thus, disturb the epigenetic pattern of RAGE signaling pathways. Abbreviations: AGEs—advanced glycation end-products; RAGE—receptor for advanced glycation end-products; S100B—S100 calcium-binding protein B; HMGB1—high-mobility group box 1; Diaph1—diaphanous related formin 1; NFKB—nuclear factor kappa-light-chain enhancer of activated B cells; STAT—signal transducer and activator of transcription; JAK—Janus-activated kinases.

Extracellular HMGB1 is a proinflammatory ligand that binds to the V domain of RAGE and thereby activates Janus kinase-signal transduction, the transcription activation (JAK/STAT) signaling pathway, and nuclear factor KB-dependent (NFKB) cytokine production (Figure 3) [12,53]. Studies have shown that cancer cells release HMGB1 [54]. Increasing amounts of HMGB1 activate the RAGE signaling pathway. Feedback loops between HMGB1 and RAGE have been observed during the progression of cancer [8,54]. We hypothesize that blocking the cross-talk between RAGE and HMGB1 may inhibit cancer progression. Researchers should consider the RAGE-HMGB1-dependent signaling pathway when searching for therapeutic targets to prevent EC progression.

### 3.2. S100B

The S100B is part of the family of small molecular weight calcium-binding proteins [8,42,55]. Our studies have shown that the amount of S100B is increased in the diabetic endometrium [6]. Most studies implicated S100B in inflammation and cancer progression. That is why this molecule is called a proinflammatory RAGE ligand and a marker of neurodegenerative diseases [12,42,56,57,58]. Studies confirmed that S100B may be considered a tumor marker [46]. Elevated serum S100B levels have already been proposed as a biomarker for lung cancer and melanoma [59,60,61]. S100B interacts with cellular tumor antigen p53 [62]. Studies have indicated that S100B may modulate p53 functions in tumor suppression and, thus, stimulate cancer progression [62,63]. Protein p53 prevents genome mutation as well as tumor formation [64]. p53 plays a prominent role in DNA repair, apoptosis control, and the cell cycle [46,64]. The data suggest that S100B may be involved in EC progression (https://www.proteinatlas.org/ENSG00000160307-S100B/pathology, accessed on 1 August 2024). However, high-throughput studies revealed that S100B does not correlate with the estrogen-negative group of cancer [46]. Yen et al. [46] have shown that in breast cancer the amount of S100B was decreased when compared to healthy tissue. This mode of S100B activity may be specific to breast cancer [46]. The interaction of S100B with RAGE deserves attention in the next steps of EC research.

### 3.3. Diaph1

Diaph1 is an intracellular ligand of RAGE. The cytoplasmatic domain of RAGE interacts with the FH1 domain of Diaph1. Diaph1 activity is strictly autoregulated by the DID domain and the terminal DAD domain. However, the FH2 domain is involved in actin polymerization and binds to profilin 1 (PFN1) [8]. The RAGE-Diaph1 signaling pathway was first identified in disorders such as diabetes, inflammation, and neurodegenerative diseases [8,12,14,65,66]. Over the last decade, Diaph1 has gained attention as a significant contributor to the pathogenesis of a few neurodegenerative disorders, such as Alzheimer’s, Parkinson’s, Huntington’s, and Creutzfeldt–Jakob’s disease, and various neurodegenerative conditions, such as diabetic neuropathy, amyotrophic lateral sclerosis, amyloid polyneuropathy, Charcot neuropathy, vasculitis neuropathy, and cancer [8,12,14,67]; however, the detailed mechanisms of the Diaph1 contribution to disorders remain unclear (https://www.proteinatlas.org/ENSG00000131504-DIAPH1/pathology, accessed on 1 August 2024).

Recent studies have shown that in animal models of neurodegenerative diseases, the pharmacological or genetic blocking of Diaph1 attenuates inflammation and Diaph1-associated perturbations [8,9,14,68,69,70,71,72]. Studies revealed that Diaph1 deletion may improve sciatic nerve regeneration in diabetic mice [8,14,68,69,70].

**Figure 4:**
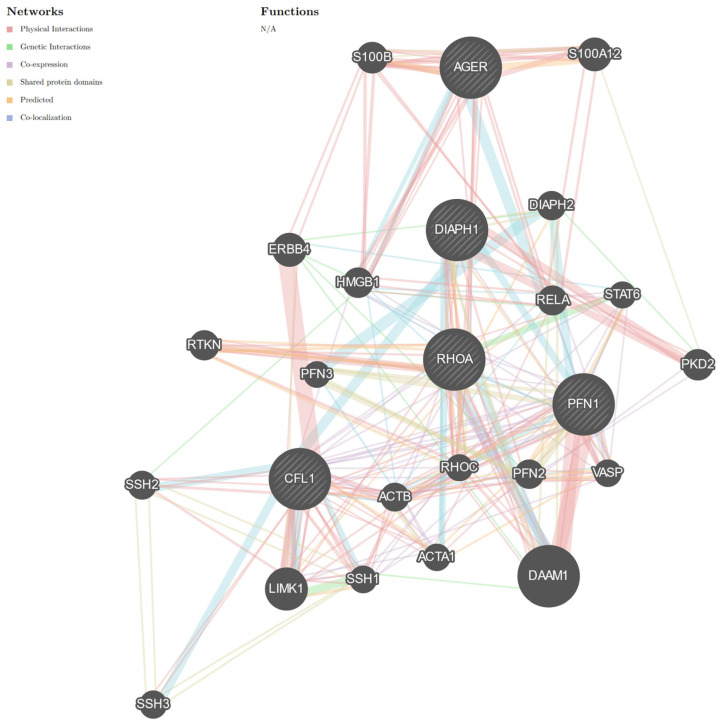
Graph representing the interaction network of *Diaph1* with proteins involved in cytoskeletal dynamics. Formin, Diaph1, is responsible for the polymerization and depolymerization of actin. It interacts with PFN1 and CFL1. Interactions between these molecules engage RhoA to interact with RAGE. Moreover, the cross-talk between RAGE and Diaph1 may play a crucial role in the remodeling of the actin cytoskeleton during the progression of EC. *AGER* (the gene encoding RAGE), *Diaph1*, *PFN1*, *CFL1*, and *RhoA* are connected in one network. The network consists of 25 genes (official symbol). STAT6 belongs to the STAT family. The STAT pathway may be dependent on the proper function of the actin cytoskeleton. The server additionally adds genes based on known interactions. Additional genes are represented by gray, unstriped circles (https://genemania.org/) [24]. The colors of the line suggest the type of interaction, that is, red—physical interactions; purple—co-expression; orange—predicted; blue—co-localization; light blue—pathway; green—genetic interactions; olive—shared protein domains. Limited to *Homo sapiens*. Abbreviations: *S100A12*—S100 calcium-binding protein A12; *AGER*—the gene encoding RAGE; *S100B*—S100 calcium-binding protein B; *Diaph1*—diaphanous-related formin 1; *Diaph2*—diaphanous-related formin 2; *PFN1*—profilin 1; *PFN3*—profilin 3; *PFN2*—profilin 2; *HMGB1*—high-mobility group box 1; *ERBB4*—Erb-b2 receptor tyrosine kinase 4; *RELA*—RELA proto-oncogene, NFKB subunit; *STAT6*—signal transducer and activator of transcription 6; *RTKN*—rhotekin; *RHOA*—ras homolog family member A; *PKD2*—polycystin 2; *SSH2*—slingshot protein phosphatase 2; *CFL1*—cofilin 1; *ACTB*—actin beta; *SSH1*—slingshot protein phosphatase 1; *RHOC*—ras homolog family member C; *SSH3*—slingshot protein phosphatase 3; *VASP*—vasodilator stimulated phosphoprotein; *LIMK1*—LIM domain kinase 1; *ACTA1*—actin alpha 1; *DAAM1*—dishevelled associated activator of morphogenesis 1. N/A—not applicable. See File S3. The interaction network using GeneMANIA was created in the publication by Zglejc-Waszak et al. [8]. These are not the same interaction networks as in the previous publication [8]. The similarity may result from the databases used by the GeneMANIA server.

RAGE-Diaph1 signaling pathways trigger the NFKB-dependent signaling cascade and, thus, proinflammatory cytokine production [8]. Nevertheless, Diaph1, as a member of the formin family, is involved in the dynamics of the actin cytoskeleton [67]. Formin, Diaph1, is involved in actin elongation as well as depolymerization through interaction with PFN1 or cofilin 1 (CFL1, Figure 4) [73]. PFN1 and CFL1 are important proteins for proper actin polymerization and elongation [73]. However, studies have indicated that high levels of PFN1 may inhibit actin elongation [74,75]. PFN1 may trigger actin elongation and the RhoA-dependent signaling cascade, and in it is the RAGE signaling pathway [76,77,78,79].

These cytoskeletal proteins also play a role in human cancer cells [80,81,82]. Recent research shows that cytoskeletal proteins play an important role in the development of human diseases [9,14,80,81,82]. Therefore, they may be a new therapeutic target in cancer treatment.

Cancer cells have a potential for growth, migration, and invasion. The proper functioning of the cytoskeleton is important in these processes [80,81,82]. Diaph1 and cytoskeletal proteins may regulate cancer cell growth and invasion by the remodeling of the actin cytoskeleton [14,80,81,82]. We observed that global deletion or silencing of Diaph1 may have an impact on actin cytoskeletal dynamics during human diseases. Data suggest that attenuation of Diaph1 can reduce F-actin polymerization in cells [39]. However, to date, we do not know the effect of Diaph1 deletion on the dynamics of the actin cytoskeleton in EC cells.

Previous studies have shown that RAGE and its proinflammatory ligands are involved in actin polymerization in breast cancer cells [83]. Moreover, the molecular complex of RAGE and its ligands may promote the migration and invasion of human breast cancer cells through actin cytoskeleton modifications [83]. We can suppose that signal transduction in the RAGE pathway occurs as a result of the proper polymerization of actin [39,84,85]. Overall, the RAGE-Diaph1 interaction and, thus, cytoskeletal dynamics may be involved in EC progression. However, further studies are necessary to clarify this mechanism.

## 4. Cancer and Diabetes

Diabetes mellitus is a set of metabolic diseases accompanied by high glucose and the development of various disorders [8,12,86]. In recent years, many investigations revealed an association between diabetes and cancer progression [86,87]. However, in this review, we do not describe all the similarities between diabetes and cancer; rather, we will focus on the role of RAGE-dependent mechanisms in diabetes and cancer.

RAGE is a protein that increases with chronic inflammation, oxidative stress, and high glucose levels. These pathological conditions are characteristic not only for diabetic complications but also for cancer. Therefore, RAGE may be involved in the process of carcinogenesis in the endometrium [42,86,87]. Contrary to appearances, cancer is a disease with a metabolic disorder. High-throughput studies have demonstrated altered expression of metabolism-related genes in EC [4]. Moreover, a disturbed metabolic mechanism is a common feature of most cancers, regardless of where they occur [88]. Neoplasm consumes very large amounts of glucose compared to healthy tissue; this phenomenon is known as the Warburg effect [4,88,89]. The hyperglycemia milieu causes an increased influx of AGEs, the activation of RAGE and its proinflammatory ligands, as well as formins involved in the dynamics of the actin cytoskeleton [8,9,14]. We observe this pathological mechanism in both diabetes complications and cancer.

Studies have shown that diabetes doubles the risk of developing liver, pancreas, and EC. However, no relationship was found between diabetes and lung cancer, but it was shown that uncontrolled hyperglycemia reduces the likelihood of prostate cancer [86,87]. The reasons for the relationship between cancer and diabetes have been the subject of debate for several decades. We are currently investigating a common signaling pathway, the RAGE-dependent pathway.

Our studies have indicated that diabetes has an impact on female reproductive systems [6]. Diabetes contributes to increased inflammation in the uterus during the estrous cycle [6]. Inflammation is a key to creating an environment for cancer progression [42,86,87]. Chronic local inflammation causes an increase in the amount of S100B and HMGB1 and, consequently, activation of the RAGE-dependent signaling pathway [6,8,90,91]. We assume that RAGE-HMGB1-S100B interactions may contribute to the pathogenesis related to diabetes and cancer [42,52,59,60,61]. Studies have indicated that silencing S100B and HMGB1 delays diabetic complications and cancer progression [42,92].

Reports have indicated that increasing oxidative stress is characteristic of cancer. Oxidative stress induces RAGE signaling pathways in cancer cells [21]. Activation of the RAGE signaling pathway increases the amount of proinflammatory cytokines [42,52,59,60,61]. During the development of cancer, there is an accumulation of negative factors: high glucose levels, inflammation, and oxidative stress. Intracorporeal homeostasis is disturbed. A high concentration of AGEs causes mutagenesis, protein misfolding, and, thus, loss of protein functions [8,9,12,13,14,15]. All these factors stimulate the proliferation and migration of cancer cells. Moreover, the RAGE pathway leads to the activation of the PI3K/Akt and NFKB-dependent signaling cascades in cancer cells. A large amount of evidence suggests that RAGE signaling pathways are related to cancer as well as diabetic perturbations [31,32].

Evidence indicates that blocking RAGE transduction may slow down cancer progression. One of the molecules that can inhibit the activity of the RAGE pathway is its variant circulating in the plasma [93,94]. It is soluble RAGE (sRAGE). sRAGE can sequester proinflammatory RAGE ligands and, thus, reduce inflammation and oxidative stress during cancer. sRAGE may be a hope for stopping cancer development [95]. Moreover, studies report that sRAGE may be used as a biomarker because low levels of circulating sRAGE may indicate the risk of cancer progression and metastasis [96]. However, further studies are necessary to clarify the role of sRAGE as a therapeutic target against cancer development.

Mangigrasso et al. [69] found a small molecule, RAGE229, that may have the ability to block the RAGE signaling pathway. RAGE229 interacts with the cytoplasmatic domain of the AGE receptor and, thus, inhibits the cross-talk between RAGE and Diaph1 [68]. The cytoplasmic domain is the site of Diaph1 binding to RAGE. Studies in a mouse model showed that the abolition of the RAGE-Diaph1 interaction may diminish inflammation and oxidative stress during perturbations [69]. To date, there is very limited knowledge on the role of Diaph1 in the progression of cancer. However, the most recent evidence indicates that Diaph1 may play a crucial role in cancer with accompanied inflammation and oxidative stress [70].

Moreover, the cross-talk between RAGE-Diaph1 is crucial during micovessel formation [15,20]. Tumor vessels are a non-physiological structure and may complicate cancer treatment [97]. Blood vessels supply cancer cells with nutrients and oxygen. These ingredients are necessary for further progression/invasion of cancer. Hence, RAGE-Diaph1 interaction may be a key factor in cancer treatment. It is well known that cancer cells promote vascularization as well as innervation. Hence, we cannot exclude the role of axonogenesis in the progression of carcinogenesis [98]. Studies have indicated that RAGE and its ligands are present in nervous cells as well as tissue [8,9,10,11,44,99]. The role of RAGE signaling during neurodegenerative diseases is well established [8,12]. The cross-talk between RAGE and Diaph1 in the neurological complications of diabetes and cancer should be the subject of further research. In this review, we have assumed that the RAGE cascade is crucial during the progression of cancer. Therefore, in the process of cancer treatment, we should look at the problem holistically. The therapy should cover many aspects key to the development of the cancer, such as inflammation, high glucose levels, oxidative stress, axonogenesis, and vasculogenesis [8,12,97,98].

Therefore, blocking the RAGE signaling pathway may be a milestone in the treatment of many pathological conditions caused by high glucose levels, oxidative stress, and inflammation. However, the switch that determines whether it will be a diabetes- or cancer-related disorder may depend on epigenetic modifications.

## 5. DNA Methylation in Endometrial Cancer (EC)

DNA methylation is one of the epigenetic modifications. Numerous studies have indicated that epigenetic modifications may be linked to gynecological malignancies [100,101]. Malfunctions in DNA methylation may play a role in the onset and progression of EC, as abnormal epigenetic patterns may be acquired throughout life. Thus, epigenetic modifications may contribute to an increased risk of gynecological malignancies [100,101].

Methylation remodeling is dependent on DNA methyltransferases, i.e., DNMT1, DNMT3a, and DNMT3b [102]. Our studies indicated that DNA methylation is maintained by the methylation complex in the uterus tissues [102,103]. The main elements of the methylation complex include DNA methyltransferases, the tripartite motif containing 28 (TRIM28), and zinc finger protein 57 (ZFP57) [103].

Briefly, studies have revealed that zinc finger protein 57 (ZFP57) recognizes methylation sites within a DNA sequence. Next, ZFP57 interacts with TRIM28, which triggers and attracts DNA methyltransferases [103]. Therefore, DNA methylation is maintained and determined by methylation complex elements, such as ZFP57, TRIM28, DNMT1, DNMT3a, and DNMT3b [100,101,102,103].

Uterine tissues are extremely sensitive to environmental factors [104]. Our studies indicated that periconceptional undernutrition affects in utero methyltransferase expression and steroid hormone concentrations in uterine flushings and blood plasma during the peri-implantation period in domestic pigs. Studies have shown that nutrition may alter the DNA methylation levels in the uterus [104]. DNA methylation regulates gene expression and, thus, the activity of proteins, as well as signaling pathways in cells/tissues. We documented that under-nutrition alters the transcriptomic profile in the endometrium as well as in the myometrium [105,106]. In this review, we would like to demonstrate alterations in DNA methylation that influence the RAGE signaling pathway.

Many serious complications have been found to be the result of RAGE-dependent epigenetic modifications [8,9,12,13,14,15]. Kan and co-workers [107] revealed that RAGE gene promoter methylation influences diabetic retinitis. They observed that DNA methylation inhibits the activity of RAGE signaling pathways [107]. Studies showed that if the RAGE pathway is not activated, it results in a reduction in proinflammatory cytokines and, thus, the elimination of inflammation [8,91,107,108,109]. Moreover, Wu et al. [108] revealed that RAGE is regulated epigenetically by an altered microenvironment.

AGEs may alter the microenvironment in a cell and influence the RAGE signaling pathways [8,19,108]. They interact with RAGE and trigger downstream signaling pathways. Studies have indicated that the molecular interaction between AGEs and RAGE increases the production of proinflammatory cytokines. Cytokines induce inflammation and, thus, change the microenvironment in the cell. Chronic inflammation triggers the RAGE signaling pathways and increases AGE levels. Therefore, we observed feedback loops between inflammation, proinflammatory cytokines, AGEs, and RAGE signaling pathways (Figure 3) [8,19,108]. Overall, if we reduce the inflammatory environment, we inhibit the RAGE signaling pathways. Therefore, we observe epigenetic regulation of the RAGE signaling pathway.

Epigenetic modifications are sensitive to microenvironment alternations [109,110]. We may observe an altered microenvironment during cancer. In the neoplasm, we may observe metabolic alterations, high glucose levels, oxidation stress, and inflammation. All these factors can change the cell microenvironment and influence the epigenetic modifications, especially DNA methylation, which is important in the activity of the RAGE signaling pathway [107,108,109].

**Figure 5:**
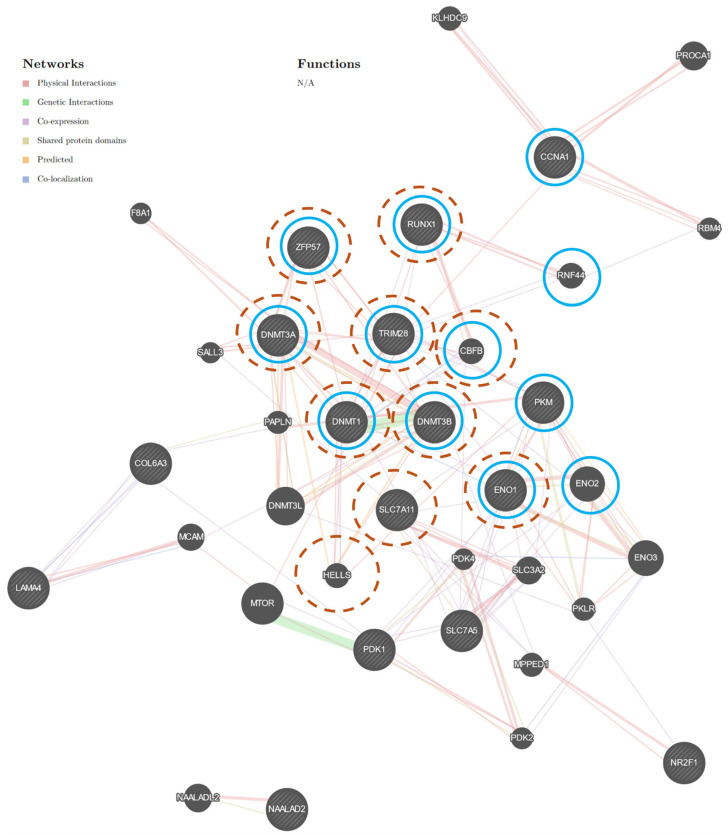
Graph representing the interaction networks of selected genes. The GeneMANIA network indicates the direct interaction between the methylation complex genes and molecules involved in metabolism during the progression of EC [4]. The in silico analysis revealed that *TRIM28* (blue circles) and *DNMT1* (orange, dashed circles) interacted with genes engaged in central carbon metabolism in cancer (hsa05230) [4], limited to *Homo sapiens* (https://genemania.org/) [24]. The colors of the line suggest the type of interaction, that is, red—physical interactions; purple—co-expression; orange—predicted; blue—co-localization; light blue—pathway; green—genetic interactions; olive—shared protein domains. Selected genes are represented by circles with stripes. Abbreviations: *MCAM*—melanoma cell adhesion molecule; *RNF44*—ring finger protein 44; *CBFB*—core-binding factor subunit beta; *HELLS*—helicase, lymphoid specific; *KLHDC9*—kelch domain-containing 9; *MPPED1*—metallophosphoesterase domain-containing 1; *PKLR*—pyruvate kinase L/R; *PAPLN*—papilin, proteoglycan-like sulfated glycoprotein; *RBM4*—RNA-binding motif protein 4; *PDK2*—pyruvate dehydrogenase kinase 2; *PDK4*—pyruvate dehydrogenase kinase 4; *F8A1*—coagulation factor VIII associated 1; *SALL3*—spalt-like transcription factor 3; *SLC3A2*—solute carrier family 3 member 2; *NAALADL2*—N-acetylated alpha-linked acidic dipeptidase-like 2; *PROCA1*—protein interacting with cyclin A1; *ENO2*—enolase 2; *ENO3*—enolase 3; *DNMT3L*—DNA methyltransferase 3-like; *MTOR*—mechanistic target of rapamycin kinase; *TRIM28*—tripartite motif-containing 28; *CCNA1*—cyclin A1; PDK1—pyruvate dehydrogenase kinase 1; *PKM*—pyruvate kinase M1/2; *ENO1*—enolase 1; *RUNX1*—RUNX family transcription factor 1; *DNMT1*—DNA methyltransferase 1, *LAMA4*—laminin subunit alpha 4, *DNMT3A*—DNA methyltransferase 3 alpha, *NR2F1*—nuclear receptor subfamily 2 group F member 1; *DNMT3B*—DNA methyltransferase 3 beta; *SLC7A5*—solute carrier family 7 member 5; *COL6A3*—collagen type VI alpha 3 chain; *SLC7A11*—solute carrier family 7 member 11; *ZFP57*—zinc finger protein 57. N/A—not applicable. See File S4.

Sidorkiewicz et al. [4] revealed that metabolic status may be involved in EC progression. The GeneMANIA [24] analysis revealed that *SLC7A11*, *SLC7A5*, *RUNX1*, *LAMA4*, *COL6A3*, *PDK1*, *CCNA1*, *ENO1*, *PKM*, *NR2F1*, *NAALAD2* [4], *DNMT1*, *DNMT3a*, *DNMT3b*, *TRIM28*, and *ZFP57* (methylation complex genes) are involved in two networks (Figure 5). We observed the direct interaction between methylation complex genes and molecules involved in metabolism during the progression of EC (Figure 5). The in silico analysis revealed that *DNMT1* directly interacts with *RUNX1*, *ENO1*, *SLC7A5*, and the cross-talk *TRIM28* with *RUNX1*, *ENO1*, *PKM*, and *CCNA1* (Figure 5). We suppose that methylation complex molecules may be involved in the regulation of the central carbon metabolism pathway (hsa05230) in EC [4]. Analysis revealed that *DNMT1* is involved in DNA methylation or demethylation, DNA modification, DNA methylation-dependent heterochromatin assembly, regulation of histone H3-K9 methylation, methylation, and the regulation of the DNA methylation-dependent heterochromatin assembly function (Figure 5). Nevertheless, *TRIM28* is engaged in the regulation of DNA methylation-dependent heterochromatin assembly and the DNA methylation-dependent heterochromatin assembly function (Figure 5). Our in silico analysis showed the methylation complex, and, thus, DNA methylation may be involved in EC (Figure 5).

Future studies on the role of DNA methylation in the activity of RAGE signaling pathways will likely accelerate our understanding of the complex signaling events in EC [111]. In the review, we emphasize the role of a holistic view of the cause of gynecological malignancies (https://www.proteinatlas.org/ENSG00000130816-DNMT1/pathology, accessed on 1 August 2024; https://www.proteinatlas.org/ENSG00000119772-DNMT3A/pathology, accessed on 1 August 2024; https://www.proteinatlas.org/ENSG00000130726-TRIM28/pathology, accessed on 1 August 2024; https://www.proteinatlas.org/ENSG00000204644-ZFP57/pathology, accessed on 1 August 2024; https://www.proteinatlas.org/ENSG00000088305-DNMT3B/pathology, accessed on 1 August 2024).

Promoter methylation cooperates with single nucleotide polymorphisms (SNPs) to modulate RAGE transcription and may alter the perturbation risks. Studies have indicated that alle-374T is associated with non-proliferative retinopathy. Moreover, studies have shown that the rs1800624 and rs1800625 alleles are correlated with ulcerative colitis. Studies have shown that the mentioned polymorphisms in the promoter activate transcription of the *RAGE/AGER* gene. We also propose that the methylation status and *RAGE* promoter genotype could jointly serve as clinical biomarkers to assist in perturbation risk assessments. However, further studies are necessary to decipher the SNPs in EC.

## 6. Conclusions

The data on the potential role of RAGE signaling pathways in EC are growing. Environmental factors such as hyperglycemia, inflammation, and oxidative stress may have an impact on the progression of EC. It is possible that RAGE signaling pathways are epigenetically controlled, particularly through DNA methylation. DNA methylation may play a crucial role in the progression of EC. These data justify further studies to fully elucidate the role of DNA methylation and RAGE signaling pathways in gynecological malignancies.

## Figures and Tables

**Table 1:** In silico analysis revealed that the gene network is involved in multiple metabolic functions.

Function	Official Symbol	Number of Genes
*pyruvate metabolic process*	*PDK1*, *PDP1*, *PDP2*, *PDPR*, *LDHA*, *PKLR*, *PGAM1*, *ENO2*, *ENO1*, *ENO3*	13
*glucose catabolic process to pyruvate*	*PKLR*, *PGAM1*, *ENO2*, *PKM*, *ENO1*, *ENO3*, *PGAM2*	7
*glycolytic process through fructose-6-phosphate*	*PKLR*, *PGAM1*, *ENO2*, *PKM*, *ENO1*, *ENO3*, *PGAM2*	7
*glycolytic process through glucose-6-phosphate*	*PKLR*, *PGAM1*, *ENO2*, *PKM*, *ENO1*, *ENO3*, *PGAM2*	7
*glucose catabolic process*	*PKLR*, *PGAM1*, *ENO2*, *PKM*, *ENO1*, *ENO3*, *PGAM2*	7
*NADH metabolic process*	*PKLR*, *PGAM1*, *ENO2*, *PKM*, *ENO1*, *ENO3*, *PGAM2*	7
*ADP metabolic process*	*PKLR*, *PGAM1*, *ENO2*, *PKM*, *ENO1*, *ENO3*, *PGAM2*, *LDHA*, *ENO4*	9
*ATP generation from ADP*	*PKLR*, *PGAM1*, *ENO2*, *PKM*, *ENO1*, *ENO3*, *PGAM2*, *LDHA*, *ENO4*	9
*NAD metabolic process*	*PKLR*, *PGAM1*, *ENO2*, *PKM*, *ENO1*, *ENO3*, *PGAM2*	7
*purine ribonucleoside diphosphate metabolic process*	*PKLR*, *PGAM1*, *ENO2*, *PKM*, *ENO1*, *ENO3*, *PGAM2*, *LDHA*, *ENO4*	9
*carbohydrate catabolic process*	*PKLR*, *PGAM1*, *ENO2*, *PKM*, *ENO1*, *ENO3*, *PGAM2*, *LDHA*, *ENO4*, *ENOSF1*	10
*purine nucleoside diphosphate metabolic process*	*PKLR*, *PGAM1*, *ENO2*, *PKM*, *ENO1*, *ENO3*, *PGAM2*, *LDHA*, *ENO4*	9
*ribonucleoside diphosphate metabolic process*	*PKLR*, *PGAM1*, *ENO2*, *PKM*, *ENO1*, *ENO3*, *PGAM2*, *LDHA*, *ENO4*	9
*nucleoside diphosphate metabolic process*	*PKLR*, *PGAM1*, *ENO2*, *PKM*, *ENO1*, *ENO3*, *PGAM2*, *LDHA*, *ENO4*	9
*glucose metabolic process*	*PKLR*, *PGAM1*, *ENO2*, *PKM*, *ENO1*, *ENO3*, *PGAM2*, *PDK1*, *SLC25A1*	9
*hexose catabolic process*	*PKLR*, *PGAM1*, *ENO2*, *PKM*, *ENO1*, *ENO3*, *PGAM2*	7
*monosaccharide catabolic process*	*PKLR*, *PGAM1*, *ENO2*, *PKM*, *ENO1*, *ENO3*, *PGAM2*	7
*hexose metabolic process*	*PKLR*, *PGAM1*, *ENO2*, *PKM*, *ENO1*, *ENO3*, *PGAM2*, *PDK1*, *SLC25A1*	9
*glycolytic process*	*PKLR*, *PGAM1*, *ENO2*, *PKM*, *ENO1*, *ENO3*, *PGAM2*	7
*ATP metabolic process*	*PKLR*, *PGAM1*, *ENO2*, *PKM*, *ENO1*, *ENO3*, *PGAM2*, *LDHA*, *ENO4*	9
*monosaccharide metabolic process*	*PKLR*, *PGAM1*, *ENO2*, *PKM*, *ENO1*, *ENO3*, *PGAM2*, *PDK1*, *SLC25A1*	9
*hexose biosynthetic process*	*SLC25A1*, *PGAM1*, *ENO2*, *ENO1*, *ENO3*, *PGAM2*	6
*regulation of sulfur metabolic process*	*PDP2*, *PDPR*, *PDP1*, *PDK1*	4
*monosaccharide biosynthetic process*	*SLC25A1*, *PGAM1*, *ENO2*, *ENO1*, *ENO3*, *PGAM2*	6
*regulation of purine nucleotide metabolic process*	*PGAM1*, *PDP2*, *PDPR*, *PDP1*, *PDK1*, *ENO1*	6
*regulation of nucleotide metabolic process*	*PGAM1*, *PDP2*, *PDPR*, *PDP1*, *PDK1*, *ENO1*	6
*thioester biosynthetic process*	*PDP2*, *PDPR*, *PDP1*, *PDK1*, *SLC25A1*	5
*acyl-CoA biosynthetic process*	*PDP2*, *PDPR*, *PDP1*, *PDK1*, *SLC25A1*	5
*acetyl-CoA metabolic process*	*PDP2*, *PDPR*, *PDP1*, *PDK1*	4
*acyl-CoA metabolic process*	*PDP2*, *PDPR*, *PDP1*, *PDK1*, *SLC25A1*	5
*carbohydrate biosynthetic process*	*SLC25A1*, *PGAM1*, *ENO2*, *ENO1*, *ENO3*, *PGAM2*	6
*regulation of fatty acid metabolic process*	*PDP2*, *PDPR*, *PDP1*, *PDK1*	4
*cellular ketone metabolic process*	*PDP2*, *PDPR*, *PDP1*, *PDK1*	4

## Supplementary Materials

The following supporting information can be downloaded at https://www.mdpi.com/article/10.3390/cancers16183192/s1, Table S1: In silico analysis revealed that the gene network is involved in multiple biological functions; File S1: Gene functions in the network. Supplementary data for Figure 1; File S2: Gene functions in the network. Supplementary data for Figure 2; File S3: Gene functions in the network. Supplementary data for Figure 4; File S4: Gene functions in the network. Supplementary data for Figure 5.
