# Supplementary material for: Role of Receptor for Advanced Glycation End-Products in Endometrial Cancer: A Review
Source: Cancers (Basel). 2024 Sep 19;16(18):3192. doi: 10.3390/cancers16183192 (PMC11430655; doi:10.3390/cancers16183192)
Supplement: Supplementary file 1 [file cancers-16-03192-s001.zip › File S1.pdf]

Function	FDR	Genes in network	Genes in genome
pyruvate metabolic process	5.0477256900470695e-17	13	120
glucose catabolic process to pyruvate	5.041941332576316e-11	7	27
glycolytic process through fructose-6-phosphate	5.041941332576316e-11	7	27
glycolytic process through glucose-6-phosphate	5.041941332576316e-11	7	27
NADH regeneration	5.041941332576316e-11	7	27
glucose catabolic process	5.595931688546885e-11	7	28
NADH metabolic process	8.475967750771954e-11	7	31
ADP metabolic process	8.475967750771954e-11	9	102
ATP generation from ADP	8.475967750771954e-11	9	100
nucleoside diphosphate phosphorylation	8.475967750771954e-11	9	101
NAD metabolic process	8.475967750771954e-11	7	31
purine ribonucleoside diphosphate metabolic process	9.500928283081697e-11	9	105
carbohydrate catabolic process	9.500928283081697e-11	10	163
purine nucleoside diphosphate metabolic process	9.50806703677266e-11	9	106
nucleotide phosphorylation	1.0767084448518332e-10	9	109
ribonucleoside diphosphate metabolic process	1.0767084448518332e-10	9	109
nucleoside diphosphate metabolic process	2.859500112182678e-10	9	122
glucose metabolic process	3.3749469782171394e-10	9	125
hexose catabolic process	1.2524384215830424e-9	7	49
monosaccharide catabolic process	4.123166888851958e-9	7	58
hexose metabolic process	4.270882754614213e-9	9	168
monosaccharide metabolic process	2.7872626709518085e-8	9	208
glycolytic process	1.1726144505704904e-7	7	94
ATP metabolic process	1.2271846911307626e-7	9	248
regulation of purine nucleotide biosynthetic process	7.129260992185925e-7	5	29
neutral amino acid transmembrane transporter activity	7.129260992185925e-7	5	29
hexose biosynthetic process	7.585082866671209e-7	6	67
regulation of nucleotide biosynthetic process	7.934957289228299e-7	5	30
regulation of sulfur metabolic process	8.117507698921346e-7	4	10
monosaccharide biosynthetic process	0.000001062719807807525	6	72
acetyl-CoA biosynthetic process from pyruvate	0.0000011919541516678838	4	11
acetyl-CoA biosynthetic process	0.000003490672432112234	4	14
regulation of purine nucleotide metabolic process	0.000006319813923384256	6	98
thioester biosynthetic process	0.000007264575758382187	5	48
acyl-CoA biosynthetic process	0.000007264575758382187	5	48
regulation of nucleotide metabolic process	0.000007264575758382187	6	101
hydro-lyase activity	0.000007862449255990296	5	49
L-amino acid transmembrane transporter activity	0.000008496437880662527	5	50
organic acid transmembrane transport	0.000013670815338884064	6	115
carboxylic acid transmembrane transport	0.000013670815338884064	6	115
purine nucleoside bisphosphate biosynthetic process	0.000022619466279085233	5	62
nucleoside bisphosphate biosynthetic process	0.000022619466279085233	5	62
ribonucleoside bisphosphate biosynthetic process	0.000022619466279085233	5	62
acetyl-CoA metabolic process	0.000031683118505805166	4	25
carbon-oxygen lyase activity	0.000032074124899833164	5	67
amino acid transmembrane transporter activity	0.000036429534008423635	5	69
purine ribonucleotide biosynthetic process	0.000039339388040932845	6	141
acyl-CoA metabolic process	0.00004644662514625086	5	73
purine nucleotide biosynthetic process	0.000048350612493134956	6	148
ribonucleotide biosynthetic process	0.000048350612493134956	6	148
lyase activity	0.000048350612493134956	6	148
ribose phosphate biosynthetic process	0.00005775055031303625	6	153
carboxylic acid transmembrane transporter activity	0.00006119300567027089	6	155
organic acid transmembrane transporter activity	0.00006239130855766887	6	156
carbohydrate biosynthetic process	0.00006605490304159608	6	158
purine-containing compound biosynthetic process	0.00009323342563131641	6	168
neutral amino acid transport	0.00011246898674767384	4	36
thioester metabolic process	0.00013713779967614606	5	94

alanine transport	0.0001547410866956381	3	10
anion transmembrane transport	0.0001967386915477776	6	193
amino acid transport	0.00023745243836534126	5	106
nucleotide biosynthetic process	0.00024149184345745295	6	201
regulation of vacuole organization	0.00025476536721776217	4	45
nucleoside phosphate biosynthetic process	0.00031064203775212177	6	211
organic anion transmembrane transporter activity	0.00033210400404607186	6	214
ribonucleoside bisphosphate metabolic process	0.000521683913052866	5	127
nucleoside bisphosphate metabolic process	0.000521683913052866	5	127
purine nucleoside bisphosphate metabolic process	0.000521683913052866	5	127
carboxylic acid transport	0.0011268892431192097	6	267
organic acid transport	0.0012099535034297082	6	271
regulation of fatty acid metabolic process	0.0014312342412799248	4	71
sulfur compound biosynthetic process	0.002116699543293243	5	171
amino acid import across plasma membrane	0.00299176657162186	3	27
secondary active transmembrane transporter activity	0.003346560509465978	5	189
L-alpha-amino acid transmembrane transport	0.007650529764730285	3	37
basolateral plasma membrane	0.008187956525796512	3	38
amino acid import	0.00944409634704563	3	40
basal part of cell	0.010808941860991609	3	42
organelle fusion	0.011692993473643776	4	124
L-amino acid transport	0.015773101481639837	3	48
amino acid transmembrane transport	0.021922767855097655	3	54
vacuole organization	0.021922767855097655	4	147
active transmembrane transporter activity	0.022292678100388135	5	288
regulation of cellular ketone metabolic process	0.026174859305093827	4	155
microvillus	0.03228319798646687	2	11
CD4-positive, alpha-beta T cell activation	0.04672863011334898	3	71
cartilage development	0.04672863011334898	3	71
regulation of alpha-beta T cell activation	0.0587365110591952	3	77
regulation of CD8-positive, alpha-beta T cell activation	0.0814957145177767	2	18
cellular ketone metabolic process	0.0814957145177767	4	212
vascular transport	0.0814957145177767	3	87
negative regulation of alpha-beta T cell differentiation	0.0814957145177767	2	18
regulation of oxidoreductase activity	0.0814957145177767	3	88
cytosol	0.0814957145177767	4	214
apical part of cell	0.08978362592515551	4	221
