# Supplementary material for: Role of Receptor for Advanced Glycation End-Products in Endometrial Cancer: A Review
Source: Cancers (Basel). 2024 Sep 19;16(18):3192. doi: 10.3390/cancers16183192 (PMC11430655; doi:10.3390/cancers16183192)
Supplement: Supplementary file 1 [file cancers-16-03192-s001.zip › File S2.pdf]

Function	FDR	Genes in network	Genes in genome
pattern recognition receptor signaling pathway	0.000014186871511132546	7	190
positive regulation of DNA-binding transcription factor activity	0.0001813346847202378	6	170
myeloid leukocyte migration	0.00018770551727132018	6	183
leukocyte migration	0.001781192754700027	6	281
regulation of I-kappaB kinase/NF-kappaB signaling	0.00774868049974758	5	203
I-kappaB kinase/NF-kappaB signaling	0.011906186789402704	5	230
toll-like receptor signaling pathway	0.015229975324275035	4	119
leukocyte cell-cell adhesion	0.015229975324275035	5	274
granulocyte migration	0.015229975324275035	4	123
glial cell activation	0.015229975324275035	3	36
leukocyte activation involved in inflammatory response	0.015229975324275035	3	33
positive regulation of defense response	0.020290588739037104	5	296
neuroinflammatory response	0.02601226813547919	3	49
regulation of monooxygenase activity	0.02601226813547919	3	50
regulation of leukocyte mediated immunity	0.02601226813547919	4	151
macrophage activation	0.042845452277915703	3	63
regulation of dendritic cell differentiation	0.042845452277915703	2	10
actin binding	0.042845452277915703	4	195
response to molecule of bacterial origin	0.042845452277915703	4	195
positive regulation of leukocyte cell-cell adhesion	0.042845452277915703	4	187
response to nicotine	0.042845452277915703	2	10
leukocyte chemotaxis	0.042845452277915703	4	190
regulation of NIK/NF-kappaB signaling	0.042845452277915703	3	68
membrane microdomain	0.051496159497018155	4	208
negative regulation of protein-containing complex assembly	0.0529965119789033	3	77
positive regulation of myeloid leukocyte mediated immunity	0.0529965119789033	2	12
positive regulation of cell-cell adhesion	0.0529965119789033	4	216
cell chemotaxis	0.0548715767105664	4	220
stress-activated protein kinase signaling cascade	0.05587090639757544	4	225
response to angiotensin	0.05587090639757544	2	13
complement binding	0.05841366780490455	2	14
superoxide anion generation	0.05841366780490455	2	15
regulation of superoxide anion generation	0.05841366780490455	2	15
mononuclear cell migration	0.05841366780490455	3	88
NIK/NF-kappaB signaling	0.05841366780490455	3	88
opsonin binding	0.05841366780490455	2	14
behavior	0.05841366780490455	4	239
regulation of oxidoreductase activity	0.05841366780490455	3	88
miRNA metabolic process	0.06500492327106479	2	16
positive regulation of leukocyte degranulation	0.06651192155335493	2	17
regulation of leukocyte cell-cell adhesion	0.06651192155335493	4	254
positive regulation of cell activation	0.06651192155335493	4	263
cellular response to interleukin-1	0.06651192155335493	3	99
multivesicular body	0.06651192155335493	2	17
positive regulation of leukocyte activation	0.06651192155335493	4	257
neutrophil migration	0.06651192155335493	3	98
dopamine metabolic process	0.06868547301735412	2	18
regulation of superoxide metabolic process	0.07211378642331749	2	19
positive regulation of protein localization to membrane	0.07211378642331749	3	105
granulocyte chemotaxis	0.07211378642331749	3	105
regulation of type I interferon production	0.07246952594992376	3	106
positive regulation of mononuclear cell migration	0.07554240536966352	2	20
integrin complex	0.07554240536966352	2	20
type I interferon production	0.08547473588625554	3	115

cognition 0.08547473588625554 3 115
positive regulation of amyloid precursor protein catabolic process 0.08681181491958355 2 22
positive regulation of cell adhesion 0.08770251706775416 4 299
response to fungus 0.09018707730077867 2 23
regulation of leukocyte adhesion to vascular endothelial cell 0.09018707730077867 2 23
actin filament capping 0.09356515220091041 2 24
protein complex involved in cell adhesion 0.09356515220091041 2 24
negative regulation of actin filament depolymerization 0.09356515220091041 2 24
