# Supplementary material for: Role of Receptor for Advanced Glycation End-Products in Endometrial Cancer: A Review
Source: Cancers (Basel). 2024 Sep 19;16(18):3192. doi: 10.3390/cancers16183192 (PMC11430655; doi:10.3390/cancers16183192)
Supplement: Supplementary file 1 [file cancers-16-03192-s001.zip › File S3.pdf]

Function	FDR	Genes in network	Genes in genome
regulation of actin filament organization	1.304145837633215e-9	10	219
regulation of actin cytoskeleton organization	2.2714133523870637e-9	10	248
actin polymerization or depolymerization	2.838282959662987e-9	9	172
regulation of supramolecular fiber organization	6.63985269249501e-9	10	296
regulation of cellular component size	1.0246958695398499e-7	9	271
actin filament polymerization	6.981098419856072e-7	7	131
negative regulation of protein-containing complex assembly	8.734096312961453e-7	6	77
actomyosin structure organization	8.734096312961453e-7	7	143
regulation of actin polymerization or depolymerization	8.734096312961453e-7	7	142
regulation of actin filament length	8.734096312961453e-7	7	146
protein polymerization	8.734096312961453e-7	8	241
regulation of actin filament bundle assembly	0.000001177547248557917	6	82
positive regulation of supramolecular fiber organization	0.0000020101016007688708	7	170
regulation of protein polymerization	0.000002891623254069396	7	181
positive regulation of cytoskeleton organization	0.0000030265414443510847	7	184
actin filament bundle assembly	0.000004217777829982134	6	106
regulation of actin filament polymerization	0.000006498904559121448	6	115
negative regulation of protein polymerization	0.000007841058325028538	5	55
actin filament bundle organization	0.000014601863360432684	6	134
regulation of stress fiber assembly	0.00001796109537162926	5	66
cell leading edge	0.000021850389646463104	7	257
stress fiber assembly	0.00002728066790648369	5	73
regulation of actomyosin structure organization	0.000041517213676134995	5	80
contractile actin filament bundle assembly	0.000060791891458506854	5	87
positive regulation of actin filament bundle assembly	0.00020836102788785734	4	44
negative regulation of supramolecular fiber organization	0.00023766560165036906	5	116
negative regulation of cytoskeleton organization	0.0002710186790242097	5	120
regulation of I-kappaB kinase/NF-kappaB signaling	0.0034936190299174165	5	203
I-kappaB kinase/NF-kappaB signaling	0.0061912267916816	5	230
actin cytoskeleton	0.011904625685403198	5	267
tissue migration	0.011904625685403198	5	266
negative regulation of organelle organization	0.012833035396853357	5	273
cell division site	0.01285775823144224	3	43
regulation of dendritic cell differentiation	0.04005621806893382	2	10
positive regulation of leukocyte cell-cell adhesion	0.04687191128415673	4	187
regulation of NIK/NF-kappaB signaling	0.04687191128415673	3	68
cytoskeleton-dependent cytokinesis	0.0586937421040864	3	74
regulation of epithelial cell migration	0.060552795838945704	4	203
positive regulation of cell-cell adhesion	0.07492775957839679	4	216
Ras protein signal transduction	0.07568821352393777	4	218
NIK/NF-kappaB signaling	0.08651058076021174	3	88
mononuclear cell migration	0.08651058076021174	3	88
