# Supplementary material for: Role of Receptor for Advanced Glycation End-Products in Endometrial Cancer: A Review
Source: Cancers (Basel). 2024 Sep 19;16(18):3192. doi: 10.3390/cancers16183192 (PMC11430655; doi:10.3390/cancers16183192)
Supplement: Supplementary file 1 [file cancers-16-03192-s001.zip › File S4.pdf]

Function	FDR	Genes in network	Genes in genome
pyruvate metabolic process	5.374804623848723e-7	8	120
glucose metabolic process	5.374804623848723e-7	8	125
DNA alkylation	0.0000020827833193788104	6	49
hexose metabolic process	0.0000028748782413392434	8	168
glucose catabolic process to pyruvate	0.0000029444890495039498	5	27
glycolytic process through fructose-6-phosphate	0.0000029444890495039498	5	27
glycolytic process through glucose-6-phosphate	0.0000029444890495039498	5	27
NADH regeneration	0.0000029444890495039498	5	27
glucose catabolic process	0.0000031821277090504315	5	28
NADH metabolic process	0.000004483464393062817	5	31
NAD metabolic process	0.000004483464393062817	5	31
monosaccharide metabolic process	0.0000051970009475525074	8	208
DNA methylation or demethylation	0.0000052110442105192446	6	72
DNA modification	0.000024501540350225327	6	94
hexose catabolic process	0.00003603769591107493	5	49
monosaccharide catabolic process	0.00008023045714190137	5	58
regulation of purine nucleotide biosynthetic process	0.0002500429816581991	4	29
regulation of nucleotide biosynthetic process	0.0002721287372575048	4	30
regulation of sulfur metabolic process	0.0006909669118510493	3	10
glycolytic process	0.0007333964249557001	5	94
acetyl-CoA biosynthetic process from pyruvate	0.0008585161204761282	3	11
ATP generation from ADP	0.0009072908224291392	5	100
nucleoside diphosphate phosphorylation	0.0009118455413387363	5	101
ADP metabolic process	0.000917690178129663	5	102
purine ribonucleoside diphosphate metabolic process	0.0010173144480803934	5	105
purine nucleoside diphosphate metabolic process	0.0010252649889262446	5	106
nucleotide phosphorylation	0.0010932110466199323	5	109
ribonucleoside diphosphate metabolic process	0.0010932110466199323	5	109
acetyl-CoA biosynthetic process	0.0013207684126295947	3	14
genetic imprinting	0.0013207684126295947	3	14
nucleoside diphosphate metabolic process	0.0017223172658478237	5	122
methylation	0.002565511412922431	6	239
macromolecule methylation	0.002565511412922431	6	239
negative regulation of oxidoreductase activity	0.0036224183351169776	3	20
regulation of gene expression, epigenetic	0.003626860459836255	5	146
hexose biosynthetic process	0.003626860459836255	4	67
monosaccharide biosynthetic process	0.0047058282080571934	4	72
carbohydrate catabolic process	0.005788927140361264	5	163
acetyl-CoA metabolic process	0.006331525014226939	3	25
neutral amino acid transmembrane transporter activity	0.009758251923958522	3	29
regulation of purine nucleotide metabolic process	0.014415845564780382	4	98
regulation of nucleotide metabolic process	0.015844279986639485	4	101
heterochromatin assembly	0.02608613124399358	3	41
regulation of vacuole organization	0.03376529699934888	3	45
heterochromatin organization	0.03527403620907592	3	46
ATP metabolic process	0.035622953291447865	5	248
thioester biosynthetic process	0.037584648954860994	3	48
acyl-CoA biosynthetic process	0.037584648954860994	3	48
hydro-lyase activity	0.03838619777970207	3	49
cytoplasmic translation	0.03838619777970207	3	49
L-amino acid transmembrane transporter activity	0.03998547070126992	3	50
purine ribonucleotide biosynthetic process	0.046948482939266595	4	141
vacuole organization	0.05350265113380004	4	147
purine nucleotide biosynthetic process	0.05350265113380004	4	148
ribonucleotide biosynthetic process	0.05350265113380004	4	148
ribose phosphate biosynthetic process	0.059708357133461384	4	153
purine nucleoside bisphosphate biosynthetic process	0.06460263211511744	3	

62
nucleoside bisphosphate biosynthetic process 0.06460263211511744 3 62
carbohydrate biosynthetic process 0.06460263211511744 4 158
ribonucleoside bisphosphate biosynthetic process 0.06460263211511744 3
62
positive regulation of heterochromatin assembly 0.06840331314022416 2 12
response to insulin 0.06874498942115491 4 163
purine-containing compound biosynthetic process 0.07351626083743906 4 168
positive regulation of heterochromatin organization 0.07351626083743906 2
13
positive regulation of chromatin assembly or disassembly 0.07351626083743906
2 13
negative regulation of histone methylation 0.07351626083743906 2 13
carbon-oxygen lyase activity 0.07351626083743906 3 67
amino acid transmembrane transporter activity 0.07827829227718905 3 69
regulation of fatty acid metabolic process 0.08157853063204966 3 71
DNA methylation-dependent heterochromatin assembly 0.08157853063204966 2
14
regulation of DNA methylation-dependent heterochromatin assembly
0.08157853063204966 2 14
acyl-CoA metabolic process 0.08732153383366204 3 73
regulation of histone H3-K9 methylation 0.09062152260703457 2 15
