# Supplementary material for: Role of Receptor for Advanced Glycation End-Products in Endometrial Cancer: A Review
Source: Cancers (Basel). 2024 Sep 19;16(18):3192. doi: 10.3390/cancers16183192 (PMC11430655; doi:10.3390/cancers16183192)
Supplement: Supplementary file 1 [file cancers-16-03192-s001.zip › Table S1.pdf]

Table S 1. *In silico* analysis revealed that the gene network is involved in multiple biological functions

Function	Official symbol	Number of genes
pyruvate metabolic process	*PDK1, PDP1, PDP2, PDPR, LDHA, PKLR, PGAM1, ENO2, ENO1, ENO3,*	13
glucose catabolic process to pyruvate	*PKLR, PGAM1, ENO2, PKM, ENO1, ENO3, PGAM2*	7
glycolytic process through fructose-6-phosphate	*PKLR, PGAM1, ENO2, PKM, ENO1, ENO3, PGAM2*	7
glycolytic process through glucose-6-phosphate	*PKLR, PGAM1, ENO2, PKM, ENO1, ENO3, PGAM2*	7
NADH regeneration	*PKLR, PGAM1, ENO2, PKM, ENO1, ENO3, PGAM2*	7
glucose catabolic process	*PKLR, PGAM1, ENO2, PKM, ENO1, ENO3, PGAM2*	7
NADH metabolic process	*PKLR, PGAM1, ENO2, PKM, ENO1, ENO3, PGAM2*	7
ADP metabolic process	*PKLR, PGAM1, ENO2, PKM, ENO1, ENO3, PGAM2, LDHA, ENO4*	9
ATP generation from ADP	*PKLR, PGAM1, ENO2, PKM, ENO1, ENO3, PGAM2, LDHA, ENO4*	9
nucleoside diphosphate phosphorylation	*PKLR, PGAM1, ENO2, PKM, ENO1, ENO3, PGAM2, LDHA, ENO4*	9
NAD metabolic process	*PKLR, PGAM1, ENO2, PKM, ENO1, ENO3, PGAM2*	7
purine ribonucleoside diphosphate metabolic process	*PKLR, PGAM1, ENO2, PKM, ENO1, ENO3, PGAM2, LDHA, ENO4*	9
carbohydrate catabolic process	*PKLR, PGAM1, ENO2, PKM, ENO1, ENO3, PGAM2, LDHA, ENO4, ENOSF1*	10
purine nucleoside diphosphate metabolic process	*PKLR, PGAM1, ENO2, PKM, ENO1, ENO3, PGAM2, LDHA, ENO4*	9
nucleotide phosphorylation	*PKLR, PGAM1, ENO2, PKM, ENO1, ENO3, PGAM2, LDHA, ENO4*	9
ribonucleoside diphosphate metabolic process	*PKLR, PGAM1, ENO2, PKM, ENO1, ENO3, PGAM2, LDHA, ENO4*	9
nucleoside diphosphate metabolic process	*PKLR, PGAM1, ENO2, PKM, ENO1, ENO3, PGAM2, LDHA, ENO4*	9
glucose metabolic process	*PKLR, PGAM1, ENO2, PKM, ENO1, ENO3, PGAM2, PDK1, SLC25A1*	9
hexose catabolic process	*PKLR, PGAM1, ENO2, PKM, ENO1, ENO3, PGAM2*	7
monosaccharide catabolic process	*PKLR, PGAM1, ENO2, PKM, ENO1, ENO3, PGAM2*	7
hexose metabolic process	*PKLR, PGAM1, ENO2, PKM, ENO1, ENO3, PGAM2, PDK1, SLC25A1*	9
glycolytic process	*PKLR, PGAM1, ENO2, PKM, ENO1, ENO3, PGAM2*	7
ATP metabolic process	*PKLR, PGAM1, ENO2, PKM, ENO1, ENO3, PGAM2, LDHA, ENO4*	9
monosaccharide metabolic process	*PKLR, PGAM1, ENO2, PKM, ENO1, ENO3, PGAM2, PDK1, SLC25A1*	9
regulation of purine nucleotide biosynthetic process	*ENO1, PDK1, PDP1, PDP2, PDPR*	5
neutral amino acid transmembrane transporter activity	*SLC7A5, SLC7A8, SLC7A11, SLC3A2, SLC7A10*	5

hexose biosynthetic process	*SLC25A1, PGAM1, ENO2, ENO1, ENO3, PGAM2*	6
regulation of nucleotide biosynthetic process	*ENO1, PDK1, PDP1, PDPR, PDP2*	5
regulation of sulfur metabolic process	*PDP2, PDPR, PDP1, PDK1*	4
monosaccharide biosynthetic process	*SLC25A1, PGAM1, ENO2, ENO1, ENO3, PGAM2*	6
acetyl-CoA biosynthetic process from pyruvate	*PDP2, PDPR, PDP1, PDK1*	4
acetyl-CoA biosynthetic process	*PDP2, PDPR, PDP1, PDK1*	4
regulation of purine nucleotide metabolic process	*PGAM1, PDP2, PDPR, PDP1, PDK1, ENO1*	6
regulation of nucleotide metabolic process	*PGAM1, PDP2, PDPR, PDP1, PDK1, ENO1*	6
thioester biosynthetic process	*PDP2, PDPR, PDP1, PDK1, SLC25A1*	5
acyl-CoA biosynthetic process	*PDP2, PDPR, PDP1, PDK1, SLC25A1*	5
hydro-lyase activity	*ENO2, ENO1, ENO3, ENO4, ENOSF1*	5
L-amino acid transmembrane transporter activity	*SLC7A5, SLC7A8, SLC7A11, SLC3A2, SLC7A10*	5
organic acid transmembrane transport	*SLC7A5, SLC7A8, SLC7A11, SLC3A2, SLC7A10, SLC25A1*	6
carboxylic acid transmembrane transport	*SLC7A5, SLC7A8, SLC7A11, SLC3A2, SLC7A10, SLC25A1*	6
purine nucleoside bisphosphate biosynthetic process	*PDP2, PDPR, PDP1, PDK1, SLC25A1*	5
nucleoside bisphosphate biosynthetic process	*PDP2, PDPR, PDP1, PDK1, SLC25A1*	5
ribonucleoside bisphosphate biosynthetic process	*PDP2, PDPR, PDP1, PDK1, SLC25A1*	5
acetyl-CoA metabolic process	*PDP2, PDPR, PDP1, PDK1*	4
carbon-oxygen lyase activity	*ENO1, ENO2, ENO3, ENO4, ENOSF1*	5
amino acid transmembrane transporter activity	*SLC7A5, SLC7A8, SLC7A11, SLC3A2, SLC7A10*	5
purine ribonucleotide biosynthetic process	*PDP2, PDPR, PDP1, PDK1, SLC25A1*	5
acyl-CoA metabolic process	*PDP2, PDPR, PDP1, PDK1, SLC25A1*	5
purine nucleotide biosynthetic process	*PDP2, PDPR, PDP1, PDK1, SLC25A1, ENO1*	6
ribonucleotide biosynthetic process	*PDP2, PDPR, PDP1, PDK1, SLC25A1, ENO1*	6
lyase activity	*ENO2, ENO1, ENO3, ENOSF1, ENO4, CLYBL*	6
ribose phosphate biosynthetic process	*PDP2, PDPR, PDP1, PDK1, SLC25A1, ENO1*	6
carboxylic acid transmembrane transporter activity	*SLC7A5, SLC7A8, SLC7A11, SLC3A2, SLC7A10, SLC25A1*	6

organic acid transmembrane transporter activity	*SLC7A5, SLC7A8, SLC7A11, SLC3A2, SLC7A10, SLC25A1*	6
carbohydrate biosynthetic process	*SLC25A1, PGAM1, ENO2, ENO1, ENO3, PGAM2*	6
purine-containing compound biosynthetic process	*PDP2, PDPR, PDP1, PDK1, SLC25A1, ENO1*	6
neutral amino acid transport	*SLC7A5, SLC7A8, SLC3A2, SLC7A10*	4
thioester metabolic process	*PDP2, PDPR, PDP1, PDK1, SLC25A1*	5
alanine transport	*SLC7A10, SLC7A8, SLC3A2*	3
anion transmembrane transport	*SLC7A5, SLC7A8, SLC7A11, SLC3A2, SLC7A10, SLC25A1*	6
amino acid transport	*SLC7A5, SLC7A8, SLC7A11, SLC3A2, SLC7A10*	5
nucleotide biosynthetic process	*PDP2, PDPR, PDP1, PDK1, SLC25A1, ENO1*	6
regulation of vacuole organization	*ENO2, ENO1, ENO3, ENO4*	4
nucleoside phosphate biosynthetic process	*PDP2, PDPR, PDP1, PDK1, SLC25A1, ENO1*	6
organic anion transmembrane transporter activity	*SLC7A5, SLC7A8, SLC7A11, SLC3A2, SLC7A10, SLC25A1*	6
ribonucleoside bisphosphate metabolic process	*PDP2, PDPR, PDP1, PDK1, SLC25A1*	5
nucleoside bisphosphate metabolic process	*PDP2, PDPR, PDP1, PDK1, SLC25A1*	5
purine nucleoside bisphosphate metabolic process	*PDP2, PDPR, PDP1, PDK1, SLC25A1*	5
carboxylic acid transport	*SLC7A5, SLC7A8, SLC7A11, SLC3A2, SLC7A10, SLC25A1*	6
organic acid transport	*SLC7A5, SLC7A8, SLC7A11, SLC3A2, SLC7A10, SLC25A1*	6
regulation of fatty acid metabolic process	*PDP2, PDPR, PDP1, PDK1*	4
sulfur compound biosynthetic process	*PDP2, PDPR, PDP1, PDK1, SLC25A1*	5
amino acid import across plasma membrane	*SLC3A2, SLC7A5, SLC7A8*	3
secondary active transmembrane transporter activity	*SLC7A5, SLC7A8, SLC7A11, SLC3A2, SLC25A1*	5
L-alpha-amino acid transmembrane transport	*SLC7A5, SLC7A8, SLC3A2*	3
basolateral plasma membrane	*SLC7A5, SLC7A8, SLC3A2*	3
amino acid import	*SLC7A5, SLC7A8, SLC3A2*	3
basal part of cell	*SLC7A5, SLC7A8, SLC3A2*	3
organelle fusion	*ENO1, ENO2, ENO3*	3
L-amino acid transport	*SLC7A5, SLC7A8, SLC3A2*	3
amino acid transmembrane transport	*SLC7A5, SLC7A8, SLC3A2*	3
vacuole organization	*ENO1, ENO2, ENO3, ENO4*	4
active transmembrane transporter activity	*SLC7A5, SLC7A8, SLC7A11, SLC3A2, SLC25A1*	5

regulation of cellular ketone metabolic process	*PDP2, PDPR, PDP1, PDK1*	4
microvillus	*SLC7A5, SLC7A8*	2
cartilage development	*RUNX1, RUNX2, RUNX3*	3
cellular ketone metabolic process	*PDP2, PDPR, PDP1, PDK1*	4
cytosol	*ENO1, ENO2, ENO3, ENO4*	4
negative regulation of alpha-beta T cell differentiation	*RUNX1, RUNX3*	2
regulation of oxidoreductase activity	*PDP2, PDP1, PDK1*	3
regulation of CD8-positive, alpha-beta T cell activation	*RUNX1, RUNX3*	2
cartilage development	*RUNX1, RUNX3, RUNX2*	3
regulation of alpha-beta T cell activation	*RUNX1, RUNX3, AGER*	3
cellular ketone metabolic process	*PDP2, PDP1, PDK1, PDPR*	4
vascular transport	*SLC7A5, SLC7A8, AGER*	3
apical part of cell function	*SLC7A5, SLC7A8, AGER, SLC3A2*	4
CD4-positive, alpha-beta T cel activation	*RUNX1, RUNX3, AGER*	3
